# Comparison of the Effects of Essential Oil Obtained from the Crude and Bran-Processed *Atractylodes lancea* on Lipopolysaccharide-Induced Inflammatory Injury of Human Colonic Epithelial Cells by Downregulating the IKK/NF-*κ*B Signaling Pathway

**DOI:** 10.1155/2021/5219129

**Published:** 2021-02-08

**Authors:** Yan Yu, Zhenqi Wu, Yu Han, Yuan Yuan, Hui Fan, Xinzhi Wei, Yongduo Yu

**Affiliations:** ^1^Liaoning University of Traditional Chinese Medicine, Shenyang 110847, China; ^2^The Affiliated Hospital of Liaoning University of Traditional Chinese Medicine, Shenyang 110847, China

## Abstract

**Background:**

*Atractylodes lancea* (AL) has been used in traditional Chinese medicine for the treatment of various diseases including digestive disorders. Ulcerative colitis (UC) is a common digestive system disease with a low cure rate and easy recurrence. However, it is still not clear whether AL is suitable for UC treatment. Currently, stir-baking with wheat bran is most commonly used to process AL. Here, we aimed to address the effects of the crude and bran-processed AL on UC in vitro and uncover the underlying mechanism based on regulating the IKK/NF-kappa B signaling pathway.

**Methods:**

Human colonic epithelial cells (HCoEpiC) were treated with lipopolysaccharide (LPS) to mimic the inflammatory injury of UC in vitro. The essential oil from crude and bran-processed AL was used to treat LPS-induced HcoEpiC cells. The cell viability was detected by an MTT assay. The levels of IL-4, IL-6, IL-8, IL-12, IL-1-*β*, TNF-*α,* and NO were determined by ELISA, and the mRNA expressions of IKK-*α*, NF-*κ*B, IL-4, IL-6, IL-8, and TNF-*α* were determined by RT-PCR. Meanwhile, the expressions of IKK-*α*, p-IKK-*α*, p-IKK-*β*, NF-*κ*B, IL-6, and IL-8 proteins were determined by Western blot.

**Results:**

The essential oil of AL, whether it was from crude or bran-processed AL, could significantly increase the viability of LPS-induced HCoEpiC cells. The treatment of AL essential oil also notably inhibited the productions of IL-6, IL-8, IL-12, IL-1-*β*, TNF-*α*, NO, p-IKK-*α*, p-IKK-*β,* and NF-*κ*B and downregulated the mRNA expressions of NF-*κ*B, IL-6, IL-8, and TNF-*α*. Meanwhile, IL-4 protein and mRNA expression were significantly stimulated by AL essential oil. Moreover, the essential oil from bran-processed AL was more effective than that from crude AL.

**Conclusion:**

Both kinds of AL essential oil had the anti-inflammatory effect on LPS-induced HCoEpiC, and the essential oil from bran-processed AL was more effective. The mechanism could be through the IKK/NF-*κ*B signaling pathway in vitro.

## 1. Introduction

Ulcerative colitis (UC) is a form of inflammatory bowel disease characterized by the intestine chronic inflammation [1]. The prevalence and incidence of UC is on the rise and is considered a global disease [2]. UC is a known risk factor for intestinal cancer, especially for colorectal cancer [3, 4]. At present, the main treatments include anti-inflammatory drugs, steroids, biologicals, immunomodulators, and so on. But, these drugs can only alleviate symptoms and prolong phases of remission. Moreover, some severe cases do not respond well to the treatments. Therefore, some alternative therapies are urgently needed [5].

The dried rhizome of *Atractylodes lancea* (AL) has been known and used as medication for a very long time. It is widely distributed in Eastern Asia. AL, which is called “Cang Zhu” in China, and is listed in the Chinese, Japanese, and Korean Pharmacopoeias. AL has been used as an important drug for the treatment of digestive disorders, rheumatic diseases, night blindness, and so on [6]. It has antitumor [7–9], antibacterial, antifungal [10], anti-inflammatory, and immunostimulative effects [11, 12]. AL is widely used in the treatment of gastrointestinal diseases [13].

Processing (*Pao Zhi*) played an important role in preparing Chinese medicinal materials because the herbal properties were changed by different processing methods. Processing can enhance the efficacy of Chinese medicine and change the trend of Chinese medicine action [14, 15]. There were more than 20 kinds of processing methods of AL in the past dynasties, such as stir-fried coke, stir-fried yellow, stir-fried charcoal, stir-fried with wheat bran, vinegar, salt, earthen, and wine. But now, most of the processed products except bran-fried products have been less used [16]. In the Chinese Pharmacopoeia, only bran-fried product of AL is listed.

As we knew, there was little information available in literature about the effect of AL on UC. The mechanism of action has poorly been understood. Furthermore, there were no reports and literature about comparing anti-inflammatory activities of the crude and processed AL on UC. Therefore, in this study, we aimed to address the effects of the crude and bran-processed AL on UC in vitro and uncover the underlying mechanism based on regulating the IKK/NF-kappa B signaling pathway.

## 2. Materials and Methods

### 2.1. Plant Materials and Chemicals

The crude AL, purchased in Bozhou city of Anhui province in China, was identified as the rhizome of *Atractylodes lancea* (Thunb.) DC. by Prof. Yanjun Zhai, working in School of Pharmacy, Liaoning University of TCM. The processed AL was prepared according to the method reported previously [14]. LPS was purchased from Beijing Solarbio Science and Technology Co., Ltd. (Beijing, China). ELISA kits were obtained from Shanghai Lianshuo Biotechnology Co., Ltd. (Ameko). PrimeScript® RT reagent kits with gDNA Eraser for real-time PCR and SYBR® Premix ExTaq™ II (TliRNaseH Plus) and ROX plus kits were all provided by TaKaRa Biotech Co. (Dalian, China). Primers for target genes were synthesized by Beijing Genomics Ins. (Beijing, China). TRIzol reagent was purchased from Invitrogen Corporation (CA, USA). 3-(4,5-Dimethylthiazol-2-yl)-2,5-diphenyl tetrazolium bromide (MTT) was purchased from Sigma Company. The following primary antibodies were used in Western blot analysis: rabbit antiinhibitor of nuclear factor kappa-B kinase-*α* (IKK-*α*) antibody (Cell Signaling; China), rabbit antiphospho inhibitor of nuclear factor kappa-B kinase-*α*/*β* (p-IKK-*α*/*β*) antibody (Cell Signaling; China), rabbit antinuclear factor kappa-B (NF-*κ*B) antibody (Cell Signaling, China), rabbit anti-*β*-actin antibody (Cell Signaling; China), rabbit antiinterleukin-6 (IL-6) antibody (Thermo Fisher; China), and rabbit antiinterleukin-8 (IL-8) antibody (Thermo Fisher; China). The secondary antibodies, HRP-labelled goat anti-rabbit IgG, were obtained from Cell Signaling Technology (China).

### 2.2. Cell Lines and Reagents

The HCoEpiC, a human normal colon epithelial cell line from American Type Culture Collection (ATCC, Manassas, VA, USA), was purchased from Guangzhou Jennio Biotech Co., Ltd. (China). The cells were cultured in DMEM (Hyclone, Logan, USA) medium supplemented with 10% fetal bovine serum, 100 U/mL penicillin, and 100 *μ*g/mL streptomycin (Gibco) at 37°C in a humidified incubator with 5% CO_2_.

### 2.3. Preparation of Essential Oils

Essential oils were extracted by steam distillation. 1000 g rhizomes of crude AL and bran-processed AL were placed in round bottom flasks, respectively. 8000 mL water was added. Distilling flasks with the volatile oil extractor and condenser were connected. The distilling flask needed to be heated at 100°C for 8 h by a heating mantle. Finally, the essential oils were collected [10]. Stock solutions were prepared by dissolving 200 mg extracts in 1 mL dimethyl sulphoxide (DMSO) and adding DMEM to 100 mL. The stock solutions were filtered with a sterile 0.22 *μ*m microporous membrane, stored at 4°C and protected from light.

### 2.4. Analysis of Essential Oil by GC-MS

100 *μ*L of essential oils was transferred into a 5 mL standard flask, and n-hexane was added to the mark. Samples were analyzed on the Agilent GC-MS system (7890B-5977 A, Agilent, USA) using HP-5 phenyl methyl silox (30 m × 0.25 mm × 0.25 *μ*m) capillary. Helium was used as carrier gas at a flow of 1 mL/min. 1 *μ*L of samples was injected in the splitless mode. The temperature program of the oven was with the initial temperature of 50°C for 3 min, raised for 10°C linearly to 290°C. Mass spectra were analyzed at 70 eV, and ion source temperature was kept at 200°C. Total ion chromatogram was created for m/*z* range 45–650. Interpretations on mass spectrum of GC-MS were performed using the database of NIST libraries (National Institute of Standards and Technology). The relative percentage amount of each component was calculated by comparing its peak area to the total area of peaks in the chromatogram.

### 2.5. Screening the Optimal Concentration of LPS by MTT

The HCoEpiCs suspensions were planted in 96-well culture plate at 1 × 10^5^ cells/well, respectively, and grown for 24 h. Different final concentrations of LPS (5, 10, and 15 *μ*g/mL) were added and incubated for 12, 24, 48, and 72 h, respectively. The suspension without LPS was used as the control. Then, 20 *μ*l MTT (5 mg/mL) was added and incubated at 37°C for an additional 2 h. The supernatants were removed, and the formazan was dissolved in 150 *μ*L DMSO for 10 min. The absorbance was recorded at 570 nm by a microplate reader (Infinite M200, TECAN, Austria). The experiment was repeated three times.

### 2.6. Cytotoxicity of Essential Oil Assessment by MTT

Cells were seeded into 96-well plates at a density of 1 × 10^5^ cells/well and cultured at 37˚C for 24 h. Essential oils from crude and bran-processed AL (0.5, 1, 10, 100, and 1000 *μ*g/mL) were added and incubated for 12, 24, 48, and 72 h respectively. The cell viabilities were measured by MTT assay.

### 2.7. Effect of Essential Oil on Cell Viability by MTT

The HCoEpiCs suspensions were planted in 96-well culture plate at 1 × 10^5^ cells/well, respectively, and cultured for 24 h. Except the control group, LPS (10 *μ*g/ml) was added. The suspension without essential oil was used as the LPS group. Different final concentrations of essential oil obtained from the crude and bran-processed AL (0.5, 1, 10, 100, and 1000 *μ*g/mL) were added and incubated for 12, 24, 48, and 72 h, respectively. The cell viabilities were measured by MTT assay. The experiment was repeated three times.

### 2.8. Determination of IL-4, IL-6, IL-8, IL-12, IL-1*β*, TNF-*α,* and NO

HCoEpiCs were seeded in flask overnight and subsequently incubated with various concentrations (1, 10, and 100 *μ*g/mL) of essential oil of AL and LPS (10 *μ*g/mL) for 24, 48, and 72 h respectively. The cell supernatants were collected (the cells were used for Western blotting analysis). The concentrations of IL-4, IL-6, IL-8, IL-12, IL-1*β*, TNF-*α,* and NO were determined by ELISA according to the manufacturer's instructions.

### 2.9. The mRNA Expressions of IKK-*α*, NF-*κ*B, IL-4, IL-6, IL-8, and TNF-*α*

The mRNA expressions of IKK-*α*, NF-*κ*B, IL-4, IL-6, IL-8, and TNF-*α* were determined by quantitative real-time PCR. The cells, incubated with or without essential oil of AL, were subsequently treated with TRIzol reagent as recommended by the manufacturer. Total RNA was isolated utilizing the TRIzol total extraction kit. The concentration of mRNA was measured by a microspectrophotometer with the ratio of OD_260_/OD_280_ > 1.8 [17]. All reverse-transcription of total RNA into cDNA was performed using the PrimeScript^®^ RT reagent kit with gDNA Eraser. Real-time qRT-PCR, which consisted of denaturation at 95°C for 3 min and 40 cycles of denaturation for 5 sec at 95°C and annealing for 1 min at 60°C, was performed in a Stratagene Mx3000p PCR system (Agilent, German). Ct values of mRNAs were analyzed by normalizing with the internal control *β*-actin. The primers for the genes are presented in Table 1.

### 2.10. Western Blotting Analysis

The expressions of IKK-*α*, p-IKK-*α*, p-IKK-*β*, NF-*κ*B, IL-6, and IL-8 proteins were determined by Western blot. Briefly, the cells were washed with PBS and harvested. The cell harvests were lysed with RIPA lysis buffer containing 1 mmol/L PMSF. The protein concentrations were determined by the BCA method. 30 *μ*g of each protein sample was separated by 10% SDS-PAGE gel and electrotransferred onto a PVDF membrane. The incubation of membranes was performed with primary antibodies (1 : 3000), including IKK-*α*, p-IKK-*α*, p-IKK-*β*, NF-*κ*B, IL-6, and IL-8, and *β*-actin antibodies overnight at 4°C. The corresponding horseradish peroxidase- (HRP-) conjugated secondary antibodies (1 : 5000) were incubated for 1 h at room temperature. The blots were visualized by electrochemiluminescence and scanned. Data were collected from three independent sets of experiment and analyzed by densitometry using Image J software.

### 2.11. Statistical Analysis

All statistical calculations were performed using SPSS 17.0 software (Chicago, USA). All data were shown as means ± standard deviation (SD). Comparisons between groups were made by one-way analysis of variance (ANOVA). It was statistically significant when *p* value of every experimental result was less than 0.05.

## 3. Results

### 3.1. GC-MS Analysis for the Compounds of Essential Oil

**Figure 1:**
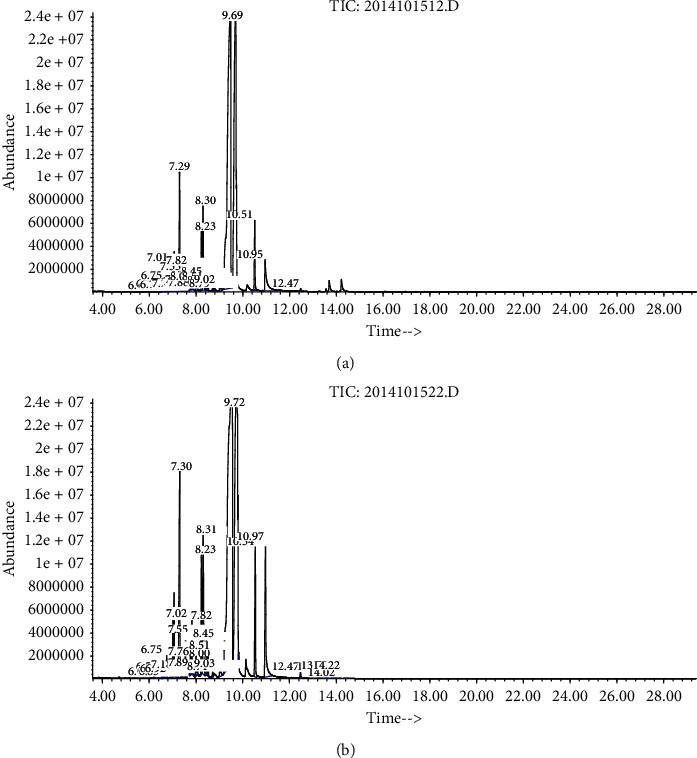
GC-MS chromatogram of essential oils of crude AL (a) and bran-processed AL (b).

Chromatograms are depicted in Figure 1 and tabulated in Table 2. In essential oils of crude and bran-processed AL, 29 and 31 compounds were detected, respectively. 4 compounds were not detected in bran-processed AL, but there were 6 new compounds in AL after processing. 12 chemical components, whose relative contents were more than 0.5%, were identified. They existed in both crude and bran-processed AL. After bran-processing, the relative contents of 5 compounds decreased, while other 7 compounds increased.

### 3.2. The Optimal Concentration of LPS

**Figure 2:**
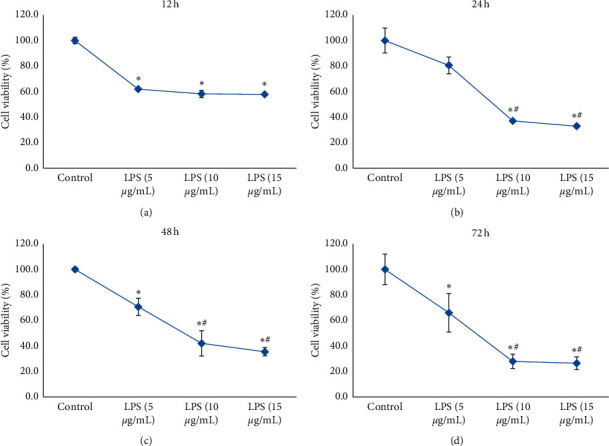
The effect of LPS on the cell viability of HCoEpiCs at 5, 10, and 15 *μ*g/mL incubated for (a) 12 (h), (b) 24 (h), (c) 48 (h), and (d) 72 (h). ^*∗*^*P* < 0.05 versus the control group, ^#^*P* < 0.05 versus the LPS (5 *μ*g/mL) group, and ^◆^*P* < 0.05 versus the LPS (10 *μ*g/mL) group.

Different final concentrations of LPS (5, 10, and 15 *μ*g/mL) were added to the cells and incubated for 12, 24, 48, and 72 h, respectively. The cell viability was detected by an MTT assay. As shown in Figure 2, LPS significantly decreased the cell viability of HCoEpiCs compared with the control group (*P* < 0.05), only except LPS (5 *μ*g/mL incubated for 24 h). Again, compared with the LPS (5 *μ*g/mL) group, the differences in LPS (10 *μ*g/mL) group or LPS (15 *μ*g/mL) group were significant (*P* < 0.05) except incubated for 12 h. But, there was no difference between the LPS (10 *μ*g/mL) group and LPS (15 *μ*g/mL) group (*P* > 0.05). So, we chose LPS (10 *μ*g/mL) to induce inflammatory injury of HCoEpiC in this study.

### 3.3. Cytotoxicity Assessment

**Figure 3:**
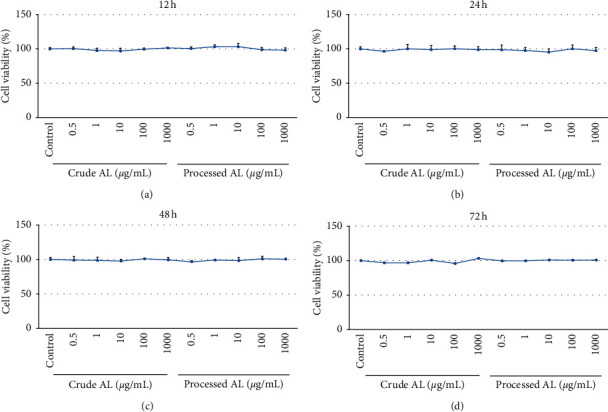
The cytotoxicity of essential oil from the crude AL and bran-processed AL on the cell viability of HCoEpiCs incubated for (a) 12 (h), (b) 24 (h), (c) 48 (h), and (d) 72 (h). ^*∗*^*P* < 0.05 versus the control group.

The results revealed that cell viabilities did not decrease among essential oil-treated groups compared with the control group (*P* > 0.05Figure 3). Essential oils did not demonstrate cytotoxicity in HCoEpiCs.

### 3.4. Effects of Essential Oil of Crude and Bran-Processed AL on Cell Viability

**Figure 4:**
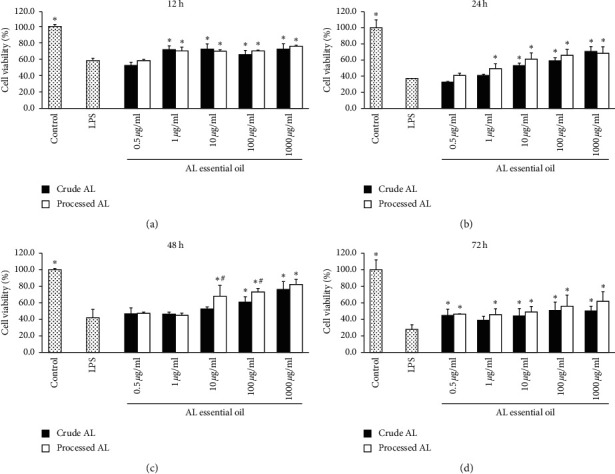
The effects of essential oil from the crude AL and bran-processed AL on the cell viability of HCoEpiCs incubated for (a) 12 (h), (b) 24 (h), (c) 48 (h), and (d) 72 (h). ^*∗*^*P* < 0.05 versus the LPS group, and ^#^*P* < 0.05 versus the crude AL group of the same concentration.

In this study, we conducted the MTT assay to evaluate the cell viability effects of essential oil from the crude and bran-processed AL on the HCoEpiCs. Essential oils (0.5, 1, 10, 100, and 1000 *μ*g/mL) were added to the cells and incubated for 12, 24, 48, and 72 h, respectively. As shown in Figure 4, LPS significantly decreased the cell viability of HCoEpiCs compared with the control group (*P* < 0.05). Compared with the LPS group, the essential oil, whether it was from crude or bran-processed AL, increased the cell viability with a few exceptions. In general, the bran-processed AL showed a better effect compared with the crude AL. The differences of bran-processed AL (10 and 100 *μ*g/mL) versus the crude AL of the same concentration were significant (48 h, *P* < 0.05).

### 3.5. Effects of AL on the Levels of IL-4, IL-6, IL-8, IL-12, IL-1*β*, TNF-*α,* and NO

**Figure 5:**
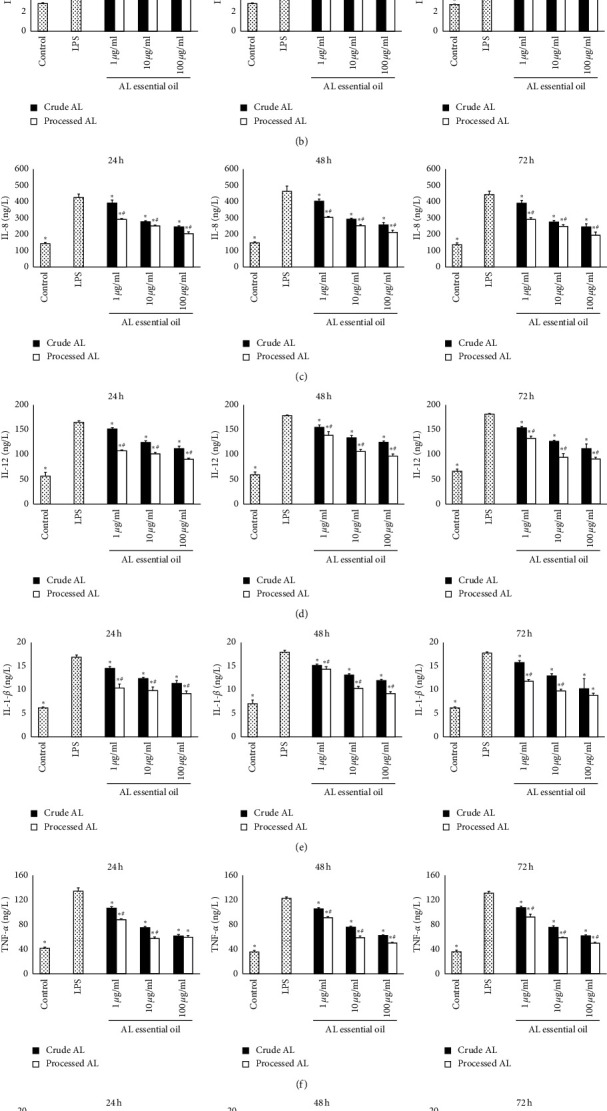
Levels of cytokines in the cell supernatants incubated for 24, 48, and 72 h were assayed by ELISA. (a) IL-4, (b) IL-6, (c) IL-8, (d) IL-12, (e) IL-1-*β*, (f) TNF-*α,* and (g) NO. Values were expressed as mean ± SD (*n* = 6). ^*∗*^*P* < 0.05 versus the LPS group, and ^#^*P* < 0.05 versus the crude AL group of the same concentration.

After treatment with the essential oil from the crude and processed AL for 24, 48, and 72 h, respectively, IL-4, IL-6, IL-8, IL-12, IL-1*β*, TNF-*α,* and NO in the cell supernatants were detected by ELISA. As shown in Figure 5, compared with the control group, the lower level of IL-4 and higher levels of IL-6, IL-8, IL-12, IL-1*β*, TNF-*α*, and NO were found in the LPS group (*P* < 0.05). The essential oil from the crude AL and bran-processed AL significantly increased the level of IL-4 (*P* < 0.05 except crude AL at the concentration of 1 *μ*g/mL incubated for 24, 48 h) and decreased the levels of IL-6 (*P* < 0.05 except crude AL at the concentration of 1 *μ*g/mL incubated for 48 h), IL-8, IL-12, IL-1*β*, TNF-*α*, and NO (*P* < 0.05) in a dose-dependent manner. Again, the differences of bran-processed AL versus the crude AL of the same concentration were significant with a few exceptions (*P* < 0.05). The processed AL showed more remarkable IL-4, IL-6, IL-8, IL-12, IL-1*β*, TNF-*α,* and NO regulation effects than the crude AL.

### 3.6. Effects of AL on the mRNA Expression of IKK-*α*, NF-*κ*B, IL-4, IL-6, IL-8, and TNF-*α*

**Figure 6:**
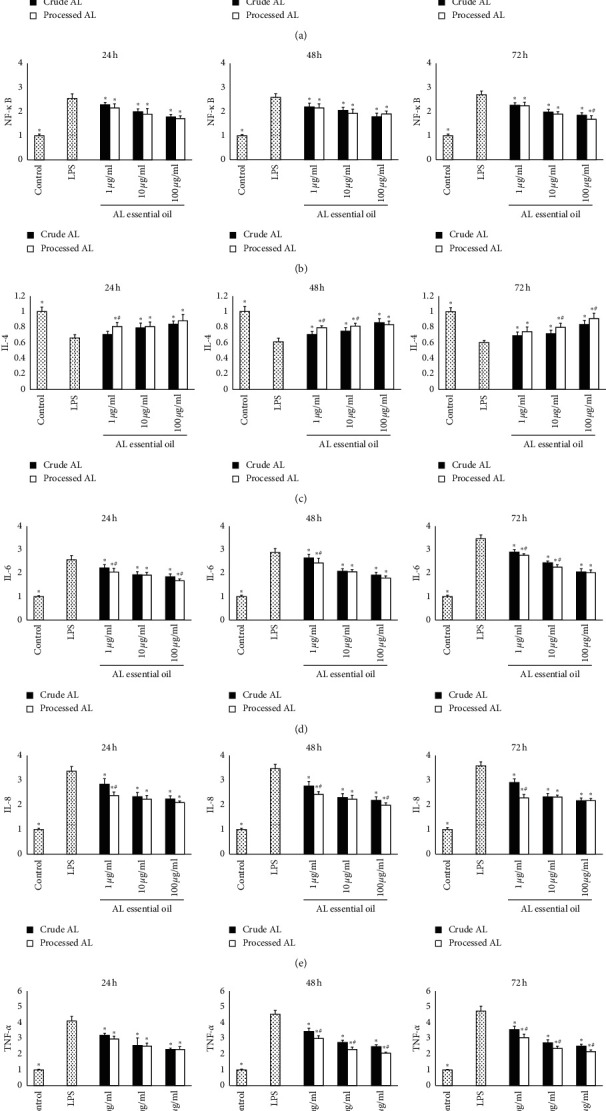
The mRNA expressions of IKK-*α* (a), NF-*κ*B (b), IL-4 (c), IL-6 (d), IL-8 (e), and TNF-*α* (f) in the cell incubated for 24, 48, and 72 h were determined by quantitative RT-PCR. Fold changes of mRNA levels of target genes related to endogenous reference *β*-actin were calculated. Data were expressed as mean ± SD (*n* = 6). ^*∗*^*P* < 0.05 versus the LPS group, and ^#^*P* < 0.05 versus the crude AL group of the same concentration.

A dissociation curve analysis of IKK-*α*, NF-*κ*B, IL-4, IL-6, IL-8, TNF-*α,* or *ββ*-actin showed a single peak. The 260/280 nm absorbance ratios were all between 1.8 and 2.0. As shown in Figure 6, compared with the control group, the lower level of IL-4 mRNA and higher levels of NF-*κ*B, IL-6, IL-8, and TNF-*α* mRNA were found in the LPS group (*P* < 0.05), but there was no difference in the level of IKK-*α* between them. The mRNA expressions of IL-4 after AL treatments were significantly higher than those in the LPS group in a dose-dependent manner. However, the mRNA expressions of NF-*κ*B, IL-6, IL-8, and TNF-*α* were significantly lower than those in the LPS group in a dose-dependent manner. In general, the processed AL showed better NF-*κ*B, IL-6, IL-8, and TNF-*α* decreasing and IL-4 increasing effects compared with the crude AL.

### 3.7. Effects of AL on the Expressions of IKK-*α*, p-IKK-*α*, p-IKK-*β*, NF-*κ*B, IL-6, and IL-8 Proteins

**Figure 7:**
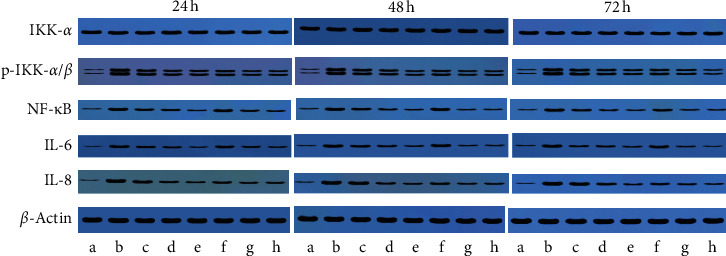
Representative images from Western blot analysis of IKK-*α*, p-IKK-*α*/*β*, NF-*κ*B, IL-6, and IL-8 in the cells (a) control, (b) LPS, (c) 1 *μ*g/ml crude AL, (d) 10 *μ*g/ml crude AL, (e) 100 *μ*g/ml crude AL, (f) 1 *μ*g/ml processed AL, (g) 10 *μ*g/ml processed AL, and (h) 100 *μ*g/ml processed AL.

**Figure 8:**
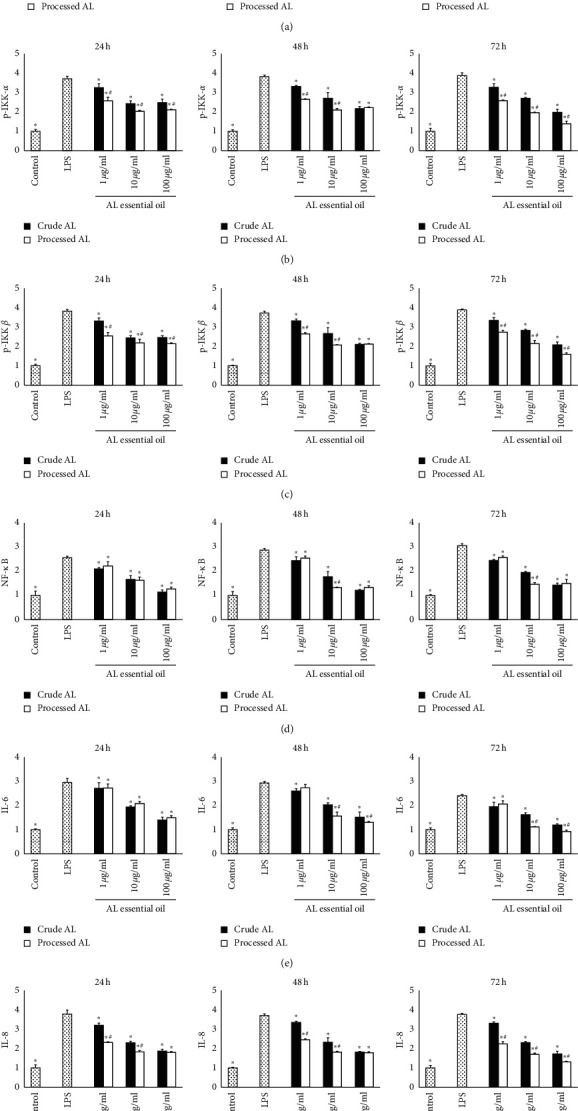
Expressions of IKK-*α* (a), p-IKK-*α* (b), p-IKK-*β* (c), NF-*κ*B (d), IL-6 (e), and IL-8(f) protein in the cells were assessed by Western blotting, as a ratio to *β*-actin. Results were expressed as mean ± SD (*n* = 3). ^*∗*^*P* < 0.05 versus the LPS group, and ^#^*P* < 0.05 versus the crude AL group of the same concentration.

After incubated with various concentrations (1, 10, and 100 *μ*g/mL) of essential oils of AL and LPS (10 *μ*g/mL) for 24, 48, and 72 h respectively, the cells were collected. The expressions of IKK-*α*, p-IKK-*α*, p-IKK-*β*, NF-*κ*B, IL-6, and IL-8 proteins were determined by Western blot. The representative images are shown in Figure 7, and the expressions of IKK-*α*, p-IKK-*α*, p-IKK-*β*, NF-*κ*B, IL-6, and IL-8 proteins are shown in Figure 8. Compared with the control group, higher levels of p-IKK-*α*, p-IKK-*β*, NF-*κ*B, IL-6, and IL-8 were found in the LPS group (*P* < 0.05). After treatment with the essential oil from AL, the levels of p-IKK-*α*, p-IKK-*β*, NF-*κ*B, IL-6, and IL-8 were lower than those in the LPS group in a dose-dependent manner. But, there was no difference in the level of IKK-*α* in all the groups. In general, the processed AL showed better p-IKK-*α*, p-IKK-*β*, NF-*κ*B, IL-6, and IL-8 decreasing effects compared with the crude AL.

## 4. Discussion

Processing, *Pao Zhi* in Chinese, is an ancient Chinese pharmaceutical technique for facilitating the use of traditional Chinese medicinal materials (TCMM) for clinical needs. Traditionally, most TCMM require a proper processing before clinical use [18]. After processing, some changes occur, which inevitably influence the associated pharmacological properties. It was traditionally considered that processing can reduce toxicity and reinforce [19]. Adjuvant medicines are frequently used in processing. Wheat bran is one of the most commonly used adjuvant medicines.

The etiology of UC is poorly known, but it is generally considered the inflammatory response to be one of the important pathogenic factors. In recent years, the role of cytokines in the development, pathogenesis, and prognosis of UC has been recognized. Studies have shown that interleukin (IL) plays an important role during the pathogenesis of UC [20]. IL-6 and IL-8 are known to be proinflammatory cytokines that possess many biological activities linked to the acute or chronic inflammatory diseases. TNF-*α* is a primary and earliest proinflammatory cytokine and regarded as a parameter of systemic and local inflammation reactions [21]. TNF-*α* and IL-6 are considered to be the markers of inflammation in patients with UC [22]. IL-12, which is a proinflammatory cytokine, plays an important role in the pathogenesis of UC [23]. IL-1*β* is a key mediator of the inflammatory and immunological response, and several biological properties may be of relevance in the pathogenesis of UC [24]. Meanwhile, IL-1*β* promotes the expressions of other inflammatory cytokines [25]. IL-4 plays a key role in maintaining intestinal immunity and inhibiting intestinal inflammation [26]. In inflammatory diseases, NO is usually considered as a potent proinflammatory factor. High NO level may promote the inflammation and cause tissue damage. There was a significant increase in the NO level after colitis induction [27, 28].

LPS, a major component of the Gram-negative bacterial cell wall [29], can trigger strong inflammatory responses and induce production of a variety of proinflammatory cytokines [30, 31]. In this study, we utilized LPS to trigger HCoEpiC inflammatory damage to compare the anti-inflammatory effects of essential oil obtained from the crude and bran-processed AL and evaluate the mechanism of anti-inflammatory action.

In the present study, first, the optimal concentrations of LPS on HCoEpiC were determined, and the results demonstrated that 10 *μ*g/mL of LPS was optimal. The essential oils did not demonstrate cytotoxicity in normal HCoEpiCs but increased the cell viability after inflammatory injury induced by LPS. In general, the processed AL showed a better effect compared with the crude AL. The levels of proinflammatory cytokines IL-6, IL-8, IL-12, IL-1*β*, TNF-*α*, and NO were increased in the LPS group. After treatment with the essential oil from the crude and bran-processed AL, lower levels of IL-6, IL-8, IL-12, IL-1*β*, TNF-*α*, and NO were observed in a dose-dependent manner. The essential oil from the bran-processed AL showed more remarkable effectiveness than that from the crude AL. However, the level of anti-inflammatory cytokine IL-4 was decreased in the LPS group. The essential oil from crude and bran-processed AL significantly increased the level of IL-4 in a dose-dependent manner. The essential oil from the bran-processed AL showed a better effect. These findings suggested that AL could decrease inflammation associated with UC, and the bran-processed AL was more effective.

The NF-*κ*B pathway is treated as the critical and classical pathway and is the primary inflammatory signal transduction pathway. Many studies have already shown that overexpression of NF-*κ*B is typical in inflamed colonic tissue [32–34]. Under normal circumstances, NF-*κ*B is bound to the inhibitory protein I*κ*B, which is normally in its inactive state. After stimulation, I*κ*B kinase (IKK) becomes phosphorylated, resulting in the phosphorylation of I*κ*B, which dissociates from NF-*κ*B and enters the nucleus to regulate the secretion and expression of inflammatory factors. The activation of IKK/NF-*κ*B, which is an essential cause of severe inflammation [35, 36], increases the secretion of the proinflammatory cytokines and induces inflammatory reactions [37, 38].

In our study, we determined the mRNA expressions of NF-*κ*B and IKK*α* by quantitative real-time PCR. The results showed that the level of NF-*κ*B was higher in the LPS group compared with the control group, and after treatment with the essential oil from AL, the level of NF-*κ*B was significantly lower than that in the LPS group in a dose-dependent manner. However, there was no difference in the level of IKK*α* in all the groups. In addition, the expressions of IKK-*α*, p-IKK-*α*, p-IKK-*β*, and NF-*κ*B proteins were determined by Western blot. Compared with the control group, higher levels of p-IKK-*α*, p-IKK-*β*, and NF-*κ*B were found in the LPS group. The levels of p-IKK-*α*, p-IKK-*β*, and NF-*κ*B were lower in the treatment groups than those in the LPS group dose-dependently. There was no difference in the level of IKK-*α* in all the groups, and this result was in coincidence with the abovementioned results. This suggested the essential oil could inhibit the phosphorylation of IKK. The essential oil from the bran-processed AL showed better effects. These results suggested that the effects of essential oil obtained from the crude and bran-processed AL on LPS-induced inflammatory injury of HCoEpiC could be attributed partly to the potent anti-inflammatory property via downregulating the IKK/NF-*κ*B signaling pathway.

Essential oil, mainly including sesquiterpenoids and polyacetylene, is the major active ingredient in AL [39]. We analyzed the composition of essential oils both from crude AL and bran-processed AL by GC-MS. The results showed that there were 6 new components and 7 components with increasing contents in processed AL. These may be the reasons the bran-processed AL had more satisfactory effects in treatment of UC than the crude AL. However, it demands further research such as isolation and fractionation to purify these compounds in order to determine their activity individually and/or in combination.

## 5. Conclusions

In summary, we evaluated the effects of the essential oil of AL on UC in vitro and compared the effects between the crude AL and bran-processed AL. We also explored the mechanism based on regulating the IKK/NF-*κ*B signaling pathway. The results suggested the essential oil obtained from both crude AL and bran-processed AL had no toxicity itself on HCoEpiCs, but it increased the cell viability of inflammatory injury HCoEpiCs, decreased the levels of proinflammatory cytokines, and increased the level of anti-inflammatory cytokine. The bran-processed AL had more satisfactory effects in treatment of inflammatory injury of HCoEpiC induced by LPS than the crude AL. Furthermore, based on the above results, we identified the anti-inflammatory effect of AL partly by downregulating the IKK/NF-*κ*B signaling pathway. Some constituents in AL changed after bran-processing. Maybe, this is why the essential oil of bran-processed AL was more effective. However, it demands further research to confirm.

## Figures and Tables

**Table 1:** Information of PCR primers.

Gene symbol	GenBank accession	Sequence （5'-3'）	
IKK*α*	NM_001278	F	GAAGGTGCAGTAACCCCTCA
		R	TGCTGAAGTCTCCCCATCTTG

NF-*κ*B	NM_003998	F	CTTAGGAGGGAGAGCCCAC
		R	TGAAACATTTGTTCAGGCCTTCC

IL-8	NM_000584	F	GAAGTTTTTGAAGAGGGCTGAGA
		R	TTTGCTTGAAGTTTCACTGGCA

IL-6	NM_000600	F	ACCCCCAGGAGAAGATTCCA
		R	TTACATGTCTCCTTTCTCAGGGC

IL-4	NM_000589	F	CTTTGCTGCCTCCAAGAACAC
		R	GCGAGTGTCCTTCTCATGGT

TNF-*α*	NM_000594	F	CCCATGTTGTAGCAAACCCTC
		R	TATCTCTCAGCTCCACGCCA

*β*-Actin	NM_001101	F	GAGCACAGAGCCTCGCCTTT
		R	TCATCATCCATGGTGAGCTGG

**Table 2:** Compounds of essential oils of crude AL and bran-processed AL.

Peak no.	Retention time	m/*z* value	Compound name	Molecular formula	% peak area	
					Crude AL	Bran-processed AL
1	6.206	196	Acetic acid, 1,7,7-trimethyl-bicyclo [2.2.1]hept-2-yl ester	C_12_H_20_O_2_	0.031	0.041
2	6.547	204	1-Ethyl-3-(propen-1-yl)adamantane	C_15_H_24_	0.088	0.129
3	6.582	204	Cyclohexene, 4-ethenyl-4-methyl-3-(1-methylethenyl)-1-(1-methylethyl)-, (3R-trans)-	C_15_H_24_	——	0.033
4	6.603	204	Tricyclo[5.4.0.0(2,8)]undec-9-ene, 2,6,6,9-tetramethyl-	C_15_H_24_	0.033	0.039
5	6.646	204	*β*-Panasinsene	C_15_H_24_	0.011	0.017
6	6.696	204	1H-Cycloprop[e]azulene, 1a,2,3,4,4a,5,6, 7b-octahydro-1,1,4,7-tetramethyl-, [1aR-(1a.alpha.,4.alpha.,4a.beta.,7b.alpha.)]-	C_15_H_24_	0.053	0.071
7	6.746	204	Benzene, 1,3,5-tris(1-methylethyl)-	C_15_H_24_	0.308	0.427
8	6.916	204	Tricyclo[5.4.0.0(2,8)]undec-9-ene, 2,6,6,9-tetramethyl-	C_15_H_24_	——	0.077
9	7.015	204	6S-2,3,8,8-Tetramethyltricyclo[5.2.2.0(1,6)]undec-2-ene	C_15_H_24_	1.085	0.736
10	7.179	204	*δ*-Selinene	C_15_H_24_	0.101	0.127
11	7.228	204	Cyclohexane,1-ethenyl-1-methyl-2-(1-methylethenyl)-4-(1-methylethylidene)-	C_15_H_24_	0.048	——
12	7.292	204	Caryophyllene	C_15_H_24_	4.346	5.930
13	7.548	204	1,4,7,-Cycloundecatriene, 1,5,9,9-tetramethyl-, Z,Z,Z-	C_15_H_24_	0.642	0.443
14	7.655	204	Naphthalene,1,2,3,4,4a,5,6,8a-octahydro-4a,8-dimethyl-2-(1-methylethenyl)-,[2R-(2.alpha.,4a.alpha.,8a.beta.)]-	C_15_H_24_	0.109	——
15	7.690	204	Naphthalene,1,2,4a,5,6,8a-hexahydro-4,7-dimethyl-1-(1-methylethyl)-, (1.alpha.,4a. alpha.,8a.alpha.)-	C_15_H_24_	0.174	——
16	7.761	204	1,3-Cyclohexadiene,5-(1,5-dimethyl-4-hexenyl)-2-methyl-, [S-(R*∗*,S*∗*)]-	C_15_H_24_	0.145	0.348
17	7.825	204	Naphthalene, decahydro-4a-methyl-1-methylene-7-(1-methylethenyl)-,[4aR-(4a. Alpha.,7.alpha.,8a.beta.)]-	C_15_H_24_	0.679	0.888
18	7.882	204	Naphthalene,1,2,3,5,6,8a-hexahydro-4,7-dimethyl-1-(1-methylethyl)-, (1S-cis)-	C_15_H_24_	0.055	0.117
19	8.003	204	Naphthalene,1,2,3,4,4a,5,6,8a-octahydro-7-methyl-4-methylene-1- 1-methylethyl)-, (1.alpha.,4a.alpha.,8a.alpha.)-	C_15_H_24_	0.402	0.559
20	8.223	204	*β*-Panasinsene	C_15_H_24_	1.616	2.219
21	8.294	204	Cyclohexanemethanol,4-ethenyl-.alpha.,.alpha.,4-trimethyl-3-(1-methylethenyl)-, [1R-(1.alpha.,3.alpha.,4.beta.)]-	C_15_H_24_	3.386	4.490
22	8.450	204	*γ*-Elemene	C_15_H_24_	0.591	0.385
23	8.514	202	*β*-Vatirenene	C_15_H_22_	0.273	0.459
24	8.713	220	Caryophyllene oxide	C_15_H_24_O	0.194	0.143
25	8.791	222	Guaiol	C_15_H_26_O	0.142	——
26	9.025	204	4,7-Methanoazulene,1,2,3,4,5,6,7,8 -octahydro-1,4,9,9-tetramethyl-, [1S-(1.alpha.,4.alpha.,7.alpha.)]-	C_15_H_24_	0.379	0.382
27	9.480	222	Hinesol	C_15_H_26_O	47.988	44.698
28	9.686	222	*β*-Eudesmol	C_15_H_26_O	31.864	27.228
29	10.510	218	2(3H)-Naphthalenone,4,4a,5,6,7,8-hexahydro-4a,5-dimethyl-3-(1-methylethylidene)-,(4ar-cis)-	C_15_H_22_O	2.561	3.337
30	10.950	182	[1,1′-Biphenyl]-4-carboxaldehyde	C_13_H_10_O	3.295	4.738
31	12.463	202	Benzene,1-methoxy-2-(1-methyl-2-methylenecyclopentyl)-	C_14_H_18_O	0.153	0.160
32	13.557	214	Anthracene, 1,2,3,4,5,6,7,8-octahydro-9,10-dimethyl-	C_16_H_22_	——	0.179
33	13.699	230	2-Acetonaphthone,1,8-dihydroxy-,6-dimethyl-	C_14_H_14_O_3_	——	0.415
34	14.025	272	Kaur-16-ene	C_20_H_32_	——	0.021
35	14.224	232	2-(2,5-Dimethoxyphenyl)cyclohex-2-enone	C_14_H_16_O_3_	——	0.411

## Data Availability

The data used to support the findings of this study are available from the corresponding author upon request.
